# Why “Animal (De)liberation” survives early criticism and is pivotal to public health

**DOI:** 10.1111/jep.12807

**Published:** 2017-08-04

**Authors:** Jan Deckers

**Affiliations:** ^1^ School of Medical Education Newcastle University Newcastle upon Tyne UK

**Keywords:** health policy, medical ethics, public health

## Abstract

In 2016, the book Animal (De)liberation: Should the Consumption of Animal Products Be Banned? was published. This article aims to engage with the critique that this book has received and to clarify and reinforce its importance for human health. It is argued that the ideas developed in the book withstand critical scrutiny. As qualified moral veganism avoids the pitfalls of other moral positions on human diets, public health policies must be altered accordingly, subject to adequate political support for its associated vegan project.

## 1 INTRODUCTION

In 2016, Ubiquity Press published my book *Animal (De)liberation: Should the Consumption of Animal Products Be Banned?* 1 I am pleased and grateful that some scholars have already published their critical reviews of the book in various places, including the articles by Laestadius^2^ and Paez^3^ in this journal issue. The aim of this article is to evaluate these reviews and to clarify and expand on some ideas that were developed in the book. In doing so, it will be emphasized that the ideas developed in the book are pivotal to people's health and that, subject to sufficient political support, they should inform public health policy.

Before embarking on this task, I would like to express that the meta‐ethical position that is adopted throughout this article, as well as in my work in general, is Pyrrhonian moral scepticism, which is the position that, whenever a claim is made that X or Y ought (not) to be done (by any moral agent in a particular situation), it is inappropriate to assert that claim with certainty.^4^ Whenever a Pyrrhonian moral sceptic claims that something is right or wrong, they are neither adopting the view that what is right or wrong is merely a matter of personal preference or taste (moral relativism) nor that its universal validity would be beyond any doubt (moral dogmatism).

## 2 A BRIEF SUMMARY OF ANIMAL (DE)LIBERATION

I would like to start by providing a very concise summary of the theory developed in the book. I argue that to address the questions whether and when the human consumption of animal products may be justifiable, one must consider not only the human interest in using animals for nutritional purposes but also a raft of other interests. These interests are:

1. An interest in avoiding the consumption of animals, including those who die naturally or accidentally, which is based on a more general animalist interest.
2. An interest in avoiding the consumption of animals who are closely related to us, which is based on a more general evolutionist interest.
3. An interest in avoiding the consumption of animal products where such consumption relies on the intentional infliction of pain, suffering, and death upon animals.
4. An interest in avoiding the consumption of animal products where such consumption relies on the intentional infliction of pain, suffering, and death upon animals who are closely related to us.
5. An interest in avoiding the consumption of animal products where such consumption relies on the intentional infliction of pain, suffering, and death upon animals with relatively great capacities for richness of experience.
6. An interest in avoiding the consumption of animal products where such consumption relies on actions that pose relatively high risks of inflicting accidental pain, suffering, and death upon animals.
7. An interest in avoiding the consumption of animal products where such consumption relies on actions that jeopardize the integrity of nature.
8. An interest in holistic health.^1^ ^(p159‐160)^

In relation to 1 and 2, it must be clarified that animalism refers to the belief that, all else being equal, we should attribute more moral significance to animals than to other organisms simply because they are more closely related to us biologically, whereas I defined our evolutionist interest, or “evolutionism,” as the belief that, all else being equal, we should attribute increasingly more moral significance to animals the more closely they are biologically related to us.^1^ ^(p80)^ However, evolutionism could also be defined more generally as the belief, encompassing both speciesism and animalism, that we should, all else being equal, attribute increasingly more moral significance to organisms the more closely they are related to us. This wider definition of evolutionism is preferable as a being's evolutionary distance to the human species is relevant to adjudicate the moral significance of all organisms, rather than that of animals only. Whereas I argued that evolutionary proximity has some moral relevance, the interest in holistic health that is referred to in item 8 is the overriding interest that moral agents should act in accordance with whenever they make any moral decision. It requires careful balancing of our interest in eating animal products, of the 7 other interests that I bring to bear on the issue, as well as of any other holistic health interests that are not included within these eight, for example, our interest in a good climate. This duty to safeguard our holistic health is also articulated in terms of a duty to minimize negative Global Health Impacts (GHIs) or to maximize positive GHIs.

It is then argued that the consumption of animal products fails to minimize negative GHIs in many situations, for example, by causing disproportionate ecological and physical health risks (chapter 1). The resulting theory is qualified moral veganism (chapter 2), the theory that vegan diets ought to be the default diets for most of the human population. It is accompanied by a political project (chapter 3), the vegan project, which strives for the implementation of qualified bans on the consumption of animal products. In chapter 4, it is argued that the different views that others, including academic scholars and slaughterhouse workers, have expressed on the question addressed in the book fail to convince, and the book's appendix considers whether vegan diets might be nutritionally sound or better than other diets.

Whilst I argued that our evolutionist interest extends even to animals who have died, the book should have explained more clearly that I believe that a nonhuman animal's relative moral significance should not be determined only by the degree to which the animal in question is related to the human species but also by whether or not the animal is alive or dead and by the animal's relative experiential complexity. All else being equal, I believe that we owe more to living animals and to animals with relatively advanced capacities to enjoy complex experiences. These other factors explain why we cannot rely only on our evolutionist interest. On account of the latter factor, I put forward the view that, for example, “killing a one‐day‐old chicken embryo may be less troubling than killing an adult mussel.”^1^ ^(p161)^

## 3 ENGAGING WITH LAESTADIUS

In relation to interest 8, Laestadius is right to point out that my theory derives from a critical reflection upon the moral importance of self‐interest. As I argued, every moral agent must “prioritise their greatest (morally relevant) interest.”^1^ ^(p7)^ The reason self‐interest is at the core of my theory relates to the fact that I adopt the view that the way in which moral agents are aware of their own interests differs fundamentally from their awareness of those of others. Each moral agent only embodies their own interests. Consequently, the private interests of others are necessarily interpreted through what I called our “individualistic bias.”^1^ ^(p67)^ Ethical theory must be mindful of this basic fact: Moral agents can only give importance to the interests of others by imagining, rather than by directly feeling, what these interests might be. This is why one can empathize with another being's hunger, for example, but one can only feel one's own. A disinterested moral evaluation of the interests of another is impossible as every perception of another's interests is mediated through, and therefore affected by the moral agent's own interests. This does not imply that one's own hunger is necessarily more important than another's. What it does imply, however, is that each moral agent has an insurmountable individualistic epistemological bias. Moral decisions about what moral agents should (not) do for moral patients are always based on the moral agent's attributions of moral patients' interests and on their interests in granting these some moral significance, rather than on what these interests actually are. In other words, moral agents inevitably make decisions by balancing their own interests.

Whereas this individualistic bias does not imply that the physical health of the moral agent should necessarily override the health interests attributed to others, Laestadius anticipates that many scholars in animal studies will take issue with my speciesism as “human interests ultimately take precedent.”^2^ Unlike our individualistic bias, this bias could be avoided as moral agents could decide to turn a blind eye to species membership. However, my book argues that they should not do so. It should be clear, however, that speciesism does not imply that peripheral human interests should be allowed to override important interests of nonhuman animals. It might, for example, be wrong to prioritize one's interest in drinking milk from nonhuman animals over one's interest in not doing so, where the latter may be based on one's moral view that drinking the milk in question would violate some important interests of nonhuman animals. Nevertheless, Laestadius is right that not all scholars in animal ethics are enamoured by my speciesist stance, an issue that I shall return to below.

I also believe that she is right in pointing out that the book could have addressed more “why moral consideration of animals is critical to the promotion of health, holistically defined.”^2^ Perhaps most controversial in this regard is the claim that our evolutionist interest is associated with some aversion towards consuming animals, which is why we may have a morally significant interest in avoiding the consumption of animals, even of those who die naturally or accidentally. In an increasingly more homogenized world, diets that include a large proportion of animal products have proliferated. Consequently, many people have (developed) appetites for the consumption of animal products. In this light, the claim that we are also predisposed to have some aversion or disgust towards the consumption of animal products and that we should avoid consuming them in many situations because of it may seem odd. For people to give any moral weight to an interest in avoiding the consumption of animals per se, however, they must recognize some morally relevant aversion or disgust towards their consumption. Whereas my moral theory does not depend on a sociological survey, my belief in it is likely to grow if I know that others share the underlying values. Conversely, it may decrease if it is found that many do not share my values.

Research will therefore need to be done to explore whether my aversion might be universally shared amongst moral agents, and whether it extends to all products derived from nonhuman animals. My hypothesis is that both questions can be answered affirmatively, but that many people fail to strike an appropriate balance between their appetite and their aversion. I can relate to this difficulty. Whereas my own dietary choices would be much more challenging if I would not have been able to benefit from the luxury of being able to eat a very varied vegan diet without much difficulty and without significant concerns over its moral adequacy, the sheer fact that some dietary choices may be hard does not provide a good reason for a failure to adhere to dietary principles. Apart from the cases mentioned in section 1.1 of the book, I provide further, more detailed cases elsewhere, however, to illustrate that there are many situations where our interest in enjoying a nutritionally balanced diet would be harmed unjustifiably if we allow our aversion towards consuming animal products to prevail.^5^ These cases also aim to respond to Laestadius's suggestion to provide more detail on what qualified bans may look like. They illustrate that the question what counts as an ethical diet cannot be addressed adequately without sensitivity to personal, social, and ecological factors, which vary significantly between different individuals.

Whereas significant controversy is likely to be generated over the claimed existence and moral relevance of our aversion towards consuming animal products, the view that zoonoses, negative ecological impacts, as well as practices that inflict pain, suffering, and death on animals undermine our health may be less controversial. Recent research has even found that the false notion that human beings somehow transcend nature and mortality may be a significant factor that causes people to support the killing of animals, and that support for this practice may in turn reinforce some sense of invulnerability. This is known as “terror management theory,” the view that people can be terrorized by their mortality and seek to manage this terror by dominating other animals and by rejecting their own animal nature.^6^ The same study found that people are less likely to support the killing of animals when their self‐esteem is boosted, underlining the crucial importance of human health for animal ethics.

I also argued that our health is jeopardized if we undermine the integrity of nature, and that this concern with nature's integrity provides an additional ground to question not only the consumption of many conventional animal products but also the development of new technologies in the farm animal sector, including the genetic engineering of animals and in vitro flesh. This unease about tampering with nature has been documented by others,^7^ even if it may not be universally shared.^8^ Whereas this does not undermine its moral validity, to facilitate moral improvement, more research will need to be done to understand why it may not be universally recognized. In spite of this concern with nature's integrity, the development of in vitro flesh and of similar alternatives to conventional animal products may be desirable to reduce the negative GHIs associated with conventional animal products.

In general, considering one's self‐interest adequately means embracing a duty to maximize positive GHIs. In relation to this duty, Laestadius rightly makes the point that Governments may not be fair in calculating GHIs, but nevertheless that “the framework could provide a useful alternative to dollar driven cost‐benefit analysis if it could be operationalized further in future work.”^2^ I agree with Laestadius here and have indeed argued in chapter three that a significant problem associated with establishing good estimates of GHIs stems from what Hardin called the tragedy of the commons, which is partly related to the lack of international policy instruments.^9^ With exception for those goods and services that should not be priced, a GHI‐based moral theory, however, need not necessarily be seen as an “alternative to dollar driven cost‐benefit analysis,”^2^ provided that the costs and benefits that feature in such an analysis reflect a comprehensive account of all the GHIs associated with particular actions.

The lack of detail that I provided in relation to what a qualified ban on the consumption of animal products might look like is related partly to my remaining uncertainties in relation to weighing up the different moral significance we should bestow upon different organisms, and partly to the complex business of evaluating the GHIs, for example, the ecological costs and benefits, of actual and potential diets. Chapter one provides no more than a summary of this complexity. As more people take the key question addressed by the book seriously, I hope that further studies and value discussions will refine GHI calculations.

Laestadius also queries the necessity of including the book's appendix, which considers the nutritional literature on vegan diets, by pointing out that the American Dietetic Association has stated already that “appropriately planned … vegan diets, are healthful.”^10^ However, I considered the appendix to be necessary to address whether this conclusion should still stand in light of more recent nutritional research as well as to engage with the many people who remain unconvinced in spite of the position by the American Dietetic Association.

## 4 ENGAGING WITH PAEZ

The most in‐depth challenge to some ideas developed in my book comes from Paez, who is unhappy about the fact that I did not discuss Whiteheadian panexperientialism in the context of its rival ontologies. Whilst I have discussed panexperientialism and its alternatives at some length elsewhere,^11^ I would like to sum up briefly why I believe that Whitehead was drawn to this view. Reflecting on Kant's^12^ idea that the “thing‐in‐itself” cannot be known, Whitehead^13^ realized that he did actually know one thing in itself rather well: He knew himself as a feeling (and sometimes also a thinking) being, where his whole body was understood as the feeling unit. He realized, however, that reductionist materialism understood bodies as spatial entities that would be utterly devoid of feelings. One problem for this ontology is how to understand the apparent reality of feelings, which is resolved by denying that they exist. Whitehead also realized that dualism rejects this solution by arguing, like reductionist materialism, that there are some things that are utterly devoid of feelings, typically including non‐living things, but that there are other things that do feel, typically including at least some living things. One problem for this ontology is how things with feelings might have emerged from those that lack them. Another issue is how our experience of ourselves as unified beings might be reconciled with the view that we are composed of 2 fundamentally distinct things, flying in the face of the commonly held belief that our minds both depend on and influence our bodies.

Faced with these problems, Whitehead decided to adopt a more parsimonious ontology that nevertheless takes the reality of our embodied experiences seriously. Whitehead started with his own experience, thinking that his mind was nothing but the integration of billions of units of feeling that were spread throughout his body and that feed into it. He then started thinking about what other things might be like, thinking not about how they were observed by him, but about what it might be like to be another individual. This led him to conclude that we should think of other things in themselves by analogy with the way in which we understand ourselves, as feeling entities. David Griffin^14^ introduced the name panexperientialism to refer to this ontology, which is the ontology that believes that all true individuals, unlike aggregates of individuals, have experiences. As my version of this ontology adopts the view that all experiences are also sentient, I suggested using the name “pan‐sentientism.”^1^ ^(p70)^ In spite of the physiological, anatomical, and behavioural data that I discuss in section 2.6 with the aim to obtain some idea of what the experiences of others might be, I admit that Kant was right: No individual is really able to know what it might be like to be another individual.

Whereas I am very comfortable with pansentientism, I am less comfortable with the task, which I nevertheless consider to be important, of ranking different entities' experiences on a scale of qualitative richness because of my individualistic bias.^1^ ^(p67)^ It is this bias that reigns supreme, which is why I disagree with Paez that adopting a panexperientialist ontology creates “ulterior problems.”^3^ No moral theory that is based on any other ontology has provided a satisfactory answer to how a moral agent might separate their own feelings and interests from the feelings and interests that they may attribute to another entity. Any moral theory that assigns moral significance to entities on the basis of their experiential capacities is therefore faced with this problem. As pansentientism argues for a continuum of these capacities, however, moral decision‐making is likely to be more troublesome than it might be for those who accept sharp dichotomies. Pansentientist qualified moral vegans may therefore be expected to have greater doubts about their moral theory compared to qualified moral vegans who adopt a dualistic ontology.

Whereas our individualistic bias is insurmountable, moral agents can reject a speciesist bias. My book argues, however, that we should not do so. A substantial point that must be clarified is that Paez wrongly concludes that my view implies that “agents act wrongly when they do *not* give greater weight to the interests of their species co‐members.”^3^ With many who have objected to speciesism I affirm, however, that “like interests should be treated alike, regardless of which species the individual with interests happens to belong to.”^1^ ^(p80)^ If what would be wrong with speciesism is the view that like interests should be given unequal moral weight, then I am not a speciesist. However, I understand speciesism to be the belief that human moral agents have a justified interest in giving special moral significance to members of our species. I write “our species” as I did not claim—pace Paez—that “extraterrestrial species” ought to prioritize their species,^3^ but that human beings ought to do so: Speciesism is used throughout the book to refer to human speciesism. Other scholars in animal ethics have ignored significant interests that moral agents must tend to when they make moral decisions. This is what my theory aims to correct, and I mentioned in the introduction that speciesism—thus conceived—is a subclass of our evolutionist interest.

Paez, however, shares neither my commitment to evolutionism nor what it would imply for human nutrition. In relation to the first point, Paez argues that adopting evolutionism would also commit me to racism, in spite of my argument against this.^1^ ^(p82‐83)^ Whereas I contended that human beings have a racist interest, I argued that this interest ought to be overridden by our greater interest in equality, which stems from the mutual recognition between people from different races agreeing that an equal world is better than a racially prejudiced world. With this, I do not argue that my antiracist interest depends on particular people from other races not being prejudiced against me. Indeed, it may be appropriate to treat them equally, in spite of the fact that they may not do so. However, mutual recognition is required in the sense that racism would not be a moral problem if nobody from another race possessed the capacity to question racism. This is why I argued that the fact that nonhuman animals, unlike people from other races, cannot make such mutual agreements with us is morally relevant. Whilst human beings and nonhuman animals cannot mutually recognize that an anti‐speciesist world might be better than a speciesist world, at least some human beings from one race and at least some from another race can recognize and agree mutually that an equal world is better than a racist world. Because of this difference, I am unpersuaded that racism is justified by my commitment to evolutionism.

Paez acknowledges as much and claims that “the victim's capacities to understand existing discrimination, rebel against it and repay in kind” are crucial in this part of my theory.^3^ This is not quite correct. What I think does the moral work here is that the moral patient is recognized to belong to a biological group that is, in spite of their evolutionary difference from the group to which I belong, sufficiently rational to allow for a mutually binding contract based on equality. When the moral patient lacks this capacity themselves, Paez claims that the only thing in my theory that might save them from discrimination is their being cared for by rational others, which Paez considers implausible. I understand why Paez comes to this conclusion, given that I wrote that “equal moral significance” should be given to “members of both races who may not possess moral agency, but are nevertheless held dearly by the imaginary parties.”^1^ ^(p83)^ I would like to clarify that the question whether or not they are “held dearly” is not morally relevant. Whilst they should be “held dearly,” regardless of their capacities, the crucial point that I would like to make is that merely their membership of another race that includes members who are recognized as being able to make a mutually binding contract based on equality with members of my race is sufficient. It is not the moral patient's capacities that do the moral work, but the fact that they are a member of a biological group that includes members who are recognized to be capable of making a mutually binding contract with us. This should prevent their victimization.

In relation to what my commitment to evolutionism would imply for human nutrition, Paez queries why we should hold on to the view that our evolutionist interest survives death,^1^ ^(p80)^ particularly since dead bodies are no more than aggregates of molecules with experiential capacities that can be deemed to be significantly inferior to those of living bodies. This is an important challenge. I argued in the book that an evolutionist interest is an interest in attributing special moral significance to those who are more closely related, regardless of whether they are either alive or dead. Whereas I remain convinced that this interest ought to survive death, one shortcoming of the book—as mentioned before—is that it did not make explicit my belief that there is a difference between the moral significance of a dead body and that of a living one. Accordingly, it may be preferable to consume a dead animal than to kill an animal first to consume them, in spite of the fact that the former may be more closely related to us. To make this discussion more concrete, I provide an example. In a situation where either is necessary to prevent malnutrition, a person would, in my view, be justified in eating a part of a whale who had died naturally, rather than to kill fish, who are more distantly related. They would, however, be wrong to eat a dead human being if the former option was not available, in spite of the fact that eating a dead human being would be preferable to killing a living one to consume them.

This also shows that evolutionism is not invoked to resolve any lingering doubt about who is the most sentient being. In spite of my doubts, I do believe quite strongly that a dead human being is insentient and that the experiences of the remaining molecules are inferior to those of a living animal. The challenge, however, remains: If we assume that physical health risks could be minimized to an acceptable level, why should we adopt the view that our evolutionist interest ought to imply a prima facie duty to refrain from eating the dead bodies of organisms who are closely related to us? Unless we adopt the view that human beings have some natural aversion towards eating the bodies of those who are closely related, I am unable to answer it. However, I also recognize that, paradoxically, we have an interest in eating animals and that sometimes our interest in “consuming an animal … ought to prevail,”^1^ ^(p160)^ which is why Paez is not quite correct to state that evolutionism demands that we regard animals as “unsuitable objects for consumption.”^2^ This is why I wrote that our “psychological health is best served by not conceiving of other animals as sources of food where our physical health does not depend on doing so.”^1^ ^(p162)^

Paez is right that the question whether the interests that I bring to bear on the morality of consuming animal products are universally shared cannot be derived from the small sample of people who were interviewed and whose views I engaged with in chapter four, which is why I wrote that “further research is needed to discuss qualified moral veganism explicitly and with more diverse groups of people.”^1^ ^(p157)^ Paez is incorrect, however, that this implies that my argument for a qualified ban is “methodologically problematic.”^3^ Whether many people agree with me may be relevant for its feasibility (and—pace Paez—I did not claim that realizing the vegan project is feasible, at least not in the short term), but it is not a necessary condition for its validity. Its validity depends, rather, on the moral evaluation of people's views. If the views of those who disagree with qualified moral veganism do not stand up to critical scrutiny, there would be nothing wrong with the vegan project or the ambition to create legal and political changes in line with qualified moral veganism. This does not take away that I remain doubtful about its validity, which is why the fact that Paez finds the ideas of the final chapter “extremely interesting” provides a powerful incentive to organize more deliberative exchanges on the theme in the future.^3^ As mentioned before, my confidence in my moral position is likely to grow if I know that others share the underlying values, but it may decrease if it is found that many do not share them.

## 5 ENGAGING WITH TORRES

Another review of my book was written by Mikel Torres Aldave.^15^ Torres does a great job in providing a chapter by chapter summary of the book, which he understands, by and large, very well. Nonetheless, some ideas have been misunderstood. In addition, he has made a number of counterarguments to my theory that fail to convince me.

Torres writes that my position is that plants possess less developed experiential capacities compared to many animals (“muchos animales”),^15^ ^(p204)^ but I actually believe that this applies to all animals. A more substantial point that must be clarified is that Torres is imprecise where he claims that I adopt the view that our dietary choices should be guided by the general rule that we should inflict as little pain and suffering as possible.^15^ This is actually merely one rule that must be balanced with other rules. Amongst these is the rule to act in accordance with our speciesist and animalist interests. It is at this point where my position differs from Torres's, who believes that speciesism is irrelevant and who doubts whether animalism is relevant.

Curiously, Torres claims that speciesism is not relevant morally because of the argument from species overlap, also known as the argument from marginal cases. This, however, does not make sense. The argument from species overlap focuses on the moral relevance of particular properties that would exist across species to counter the moral theory that different species deserve differential moral significance on the basis of each species possessing unique properties. Speciesism, however, does not need to be based on the view that species have unique properties. My commitment to speciesism is merely based on the view that the fact that individuals can be more or less closely related to one another genealogically matters morally. This flaw in Torres's understanding may have been fostered by his reading of Garner's work, who makes the same error where he claims that speciesism implies “that because an anencephalic infant is a human being … she has the same capacities as nonmarginal human beings.”^16^ Whereas Garner questions rightly whether the concept of “marginal” might be “offensive” when it is used in this context,^16^ ^(p177)^ a debate that I shall not pursue here, for the purpose of this article, I merely stress that speciesism implies only that anencephalic human beings deserve more moral significance compared to members of other species, rather than that they have the same capacities as other human beings.

An anti‐speciesist position cannot explain why it is, in my view, more problematic to eat a human being who died naturally than to eat nonhuman organisms who might need to be killed to be turned into food. An anti‐animalist position cannot explain either why, in my view, vegans may not be immoral by refusing to eat animals who die naturally or accidentally, in spite of the fact that their refusal may, all else being equal, cause more pain, suffering, and death. Vegans who oppose animalism also run into trouble in situations where they refuse to eat animals who might either have been anaesthetized before being killed or whose deaths were relatively painless compared to the numerous animals who were carved up on the land after it had been ploughed to provide their vegetables. This contrast is particularly stark when we compare the killing of one large mammal, such as a cow, with the thousands of deaths of the much smaller organisms that vegans are responsible for by their refusal to eat a cow.

Regarding this last example, it might be countered that the balance may tilt the other way if we factor into the equation the pain, suffering, and deaths that cows cause by their walking and grazing. However, I believe that there is a significant moral difference between our responsibility for the pain, suffering, and deaths inflicted on the organisms affected by our tillage and cultivation of the land and our responsibility for the pain, suffering, and deaths that result from our decision to allow a cow to graze. Allowing animals to live, even if this necessarily causes others to be killed, is not the same as killing animals. This is why ploughing the land poses a moral problem, whereas allowing a cow to continue living does not. Whilst farmers may have duties to limit the breeding of cows and to limit the negative impacts of grazing cows upon others, I would argue that the negative impacts that the grazing of a cow, per se, imposes upon others do not pose a moral problem. This is why the pain, suffering, and deaths that cows cause by grazing per se should not be entered into the moral equation when we make dietary decisions. The anti‐animalist vegan, therefore, has a real problem: How can a diet be justified if it imposes more units of what they consider to be morally relevant harm compared to another diet? Perhaps they might counter that the life of one cow is worth much more than the lives of the thousands of animals who are affected negatively by the plough. Whereas I accept that some lives matter more than others, I remain unconvinced, particularly if we consider that the thousands of other lives that are lost through ploughing include other mammals, for example, mice, rats, voles, and moles.

I would also question what anti‐animalist vegans might one day be obliged to do if the human capacity to anaesthetize animals had improved to such an extent that it would not impact negatively (due to the administration of toxic substances) upon the healthiness of eating their flesh afterwards. Rather than to eat plants, there may then be situations where they would be obliged to eat animals, at least if physical health risks could be minimized, if we remained ignorant of how we might anaesthetize plants, and if the relatively greater disvalue associated with the loss of the animal's life could be assumed to be insufficiently great to tilt the balance the other way. It might, of course, be countered that plants are insentient, which is the position that Torres adopts, but this is contested by some panexperientialists. The implication is that, in some situations, panexperientialist vegans would only be able to stick to a vegan diet if they adopted my moral theory.

It is also partly because of my commitment to animalism that Torres is correct to say that my position regards the production of in vitro flesh, in principle, as immoral. However, as animalism is no more than one interest amongst many others, I also argued that its production and consumption may be the lesser evil compared to the production and consumption of conventional animal products. Part of the problem with in vitro flesh is that it is derived from animals, thus clashing with our animalist interest, but the reason why I might nevertheless welcome its large‐scale production—where my cautious stance relates to current uncertainties about how exactly this might be done, and therefore to uncertainties about its GHIs—stems from my greater concerns with the consumption of conventional animal products and my realization that there is great social resistance to the adoption of vegan diets. For people who refrain from adopting vegan diets for justifiable reasons, as well as for those who cannot be compelled to adopt such diets in situations where they nevertheless ought to do so, the consumption of in vitro flesh may turn out to be preferable to the consumption of conventional animal products.

In spite of my recognition of our social reality, Torres is right to conclude that I favour the third political strategy, the vegan project, which was defined as “the ambition to create international and national laws to introduce ... a qualified ban” on the consumption of animal products.^1^ ^(p115)^ However, in spite of my contention that we should focus on the vegan project, this focus is not meant to exclude the 2 other strategies that I outline in the book, notably education and the adoption of better pricing mechanisms. In relation to the second strategy, however, it must be emphasized that the existence of noncapitalist economies is not the only problem, but also my concern with the ideology that all moral issues can be tackled by the adoption of appropriate pricing mechanisms. The book argues that many animal products simply ought not to be consumed, regardless of the price that one might ask for them.

This is another point where Torres differs, who argues that, as a political liberalist, he values freedom a great deal. This is why he cannot accept the third strategy. Rather, perhaps inspired by Rawls—arguably the most famous defender of political liberalism—he claims that the state ought to be reasonably neutral towards different conceptions of the good.^17^ This is where his position runs into problems as Torres also favours a prohibition on the cruellest forms of animal abuse. How could this be enacted by a supposedly neutral state? There is no view from nowhere, which is why even the state must adopt particular values that are not necessarily shared by everyone. I argue in chapter three that, since I am not a dictator and since I think it would be wrong for me to impose my values on others, a qualified ban on the consumption of animal products would only be legitimate if it was supported by sufficient people who, acting within a just political system, justifiably impose their values on those who disagree.^1^ ^(p115‐116)^ There may not be sufficient people to justify political change at the present time. However, this does not imply that, “morally” (“moralmente”), the vegan project would not be defensible.^15^ ^(p210)^ It is not inconsistent to adopt the view that everyone ought to act in a particular way and the view that nobody ought to be compelled by the state to do so as long as there is insufficient support for the former view.

The political abolition of slavery presents a different example that shows that it can be right to focus on the abolition of certain practices, rather than on their improvement. Whereas the better treatment of slaves may have been welcomed by early defenders of the abolition of slavery, I believe that they were right to insist on abolition, in spite of the fact that their views were not supported by the political majority. It does not seem to be appropriate to defend liberty where that liberty is being used to perpetuate acts that one considers to be immoral, however great one's doubts may be about what is (im)moral. As Torres's defence of liberty appears to have been inspired by Rawls, it is worth pointing out that, whilst Rawls included “freedom of thought” within his defence of the liberty principle, he did not include the freedom to act in accordance with one's thoughts.^17^ ^(p61)^

Whereas Torres does not explicitly refer to Rawls to support his stance, his position seems to have been influenced a great deal by the work of Garner as he claims that this scholar would have “a more appropriate theory for a liberal society” (“una teoría … más apropiada para una sociedad liberal”).^15^ ^(p210)^ The theory in question recognizes that nonhuman animals “are due much more” than a right “not to have suffering inflicted on them by humans,” but that nonhuman “animal advocates ought to direct their attention” to the goal of “eradicating the suffering of animals” as asking more than that would imply that one expects people to be “saints.”^16^ ^(p166‐168)^ While I am at one with Garner that the suffering that we impose upon other animals poses a moral issue, I also argued that it is inappropriate to seek to eradicate it. More importantly, I argued that significant political change for the better in relation to the consumption of animal products can only be expected if people balance the whole gamut of interests that are at stake in relation to the consumption of animal products appropriately, rather than focus merely on our interest in the limitation of suffering. Demanding that moral agents act in accordance with whichever interest deserves moral priority does not imply that one demands people to be saints. It merely demands that, in every situation, they tend to their most significant duty or, to put it differently, to the interest that ought to prevail. Whereas I agree with Garner that a “nonideal theory should focus on the most urgent injustices,”^16^ ^(p19)^ Torres's appeal to liberty fails to undermine the case for political change to tackle less urgent or minor injustices.

In the name of liberty, one could also defend that the state should allow those who autonomously wish to ruin their health the right to do so, at least where it does not harm others, which is the position that Torres takes. I agree with him on this point, but—contrary to what Torres claims^15^ ^(p209)^—it does not undermine the central claim of the book: That moral agents have an unconditional duty to maximize their health.^1^ ^(p6)^ If one believes that this duty might clash with other values, for example, the value of liberty, this may seem problematic. However, if the concept of health ought to be understood holistically, it includes one's psychological health, which includes a concern with liberty. For moral agents, what is paramount in safeguarding one's holistic health is to protect one's moral health. Such health cannot be achieved if values that ought to be prioritized are subordinated to other values. As Torres claims, many people might argue that restricting one's liberty when making dietary choices undermines their health. However, I argue at length that their conception of health is problematic when their freedom to choose what to eat is exercised in ways that override morally important factors that ought to be prioritized. As Torres does not provide any examples of situations where it might be argued that one's interest in liberty ought to trump the other interests that I argue should be prioritized in relation to various dietary scenarios, I conclude that my thesis stands firm: Our duty to care for our health demands that we abstain from the consumption of animal products in situations that most people are confronted with at our present time.

## 6 ENGAGING WITH MANCILLA

I have also provided examples of situations where people should not abstain, which is why Mancilla is incorrect to state, in her encyclopaedic article on “veganism,” that my position stipulates that “veganism is the best dietary choice if we care about the environment, i.e. if we care about the health of the planet as a whole.”^18^ Veganism should not be recommended as the best choice for the many people who would, if they committed to veganism, experience significant hardship, for example, due to their experiencing great difficulties in finding or purchasing the variety of plant‐based foods that must be consumed to maintain good health. Whereas the health of some organisms might be improved by the early deaths of people who, in spite of their inability to live healthy lives on vegan foods, refuse to consume animal foods, my theory does not demand such sacrifices to promote planetary health. Additionally, and relatedly, it is also important to emphasize that, whilst my global health concern relates to a concern with the health of all biological individuals, I do not adopt the view that “the planet as a whole” is an individual that could be either healthy or unhealthy.

Mancilla refers to my position—somewhat inaccurately, but understandably in light of the general gist—as “an innovative defense of veganism,” before discussing 3 counterarguments against “veganism.”^18^ She is right to suggest that veganism fails to minimize harm in some situations, but this first counterargument (the “minimize harm principle” challenge) does not apply to qualified moral veganism as it demands that we consume animal products in situations where not doing so would produce more harm. Because of my commitment to animalism, however, eating animal products may produce more harm compared to not doing so even in situations where this is only so because of the psychological harm associated with consuming animal products. For this reason, those who adopt my theory of qualified moral veganism have, in sharp contrast to other dietary theories that ignore evolutionism, one further reason why it may be appropriate to abstain from consuming animal products in some situations. When they face the “minimize harm principle” challenge, they have an additional defence.

Mancilla's second counterargument, which she encountered in the work of Lestel,^19^ amongst others, is summarized as the view that we should “turn meat‐eating into a ceremony” and “embrace the cruelty embedded in life” as not doing so would mean that we “see ourselves as superior to all other beings” and deny “our own animality.”^18^ This, however, is a non sequitur. Adopting qualified moral veganism implies neither that we affirm human superiority nor that we deny that we are animals. The view that we should bestow more moral significance upon human beings does not imply that human beings are superior. As I do not believe it is possible to substantiate claims about absolute superiority, claims about superiority and inferiority prompt the question: Inferior or superior in relation to what? It is not inconsistent to adopt both the view that human beings are inferior to many other animals, for example, horses, by virtue of the fact that we need to educate ourselves much longer to flourish relatively independently, and the view that we ought to value human lives more. It is not clear either how a failure to embrace cruelty might imply a denial of one's animality. However, what separates qualified moral veganism from many other theories that have been developed to promote veganism is the clear recognition—highlighted in section 3.5.3 of the book—that any dietary regime, vegan or otherwise, inflicts a great deal of pain, suffering, and death.

The third counterargument identified by Mancilla is the (supposedly feminist) critique that veganism should not be seen as the morally correct choice, but as “one choice among many others depending on individual and social circumstances.”^18^ Qualified moral veganism does not fall prey to this critique either, however, as it does not identify veganism as the morally correct choice for those for whom veganism would be “nutritionally inadequate … culturally alien … or economically prohibitive.”^18^

Whereas it is not entirely clear to what extent Mancilla embraces these counterarguments, a significant shortcoming of her article is that it does not mention that none undermine qualified moral veganism.

## 7 CONCLUSION

In this article, I provided a brief sketch of the key ideas developed in my book, *Animal (De)liberation*, and explored several challenges that have been raised against it. I argued that both qualified moral veganism and the vegan project survive critical scrutiny. As vegan diets are pivotal to public health in many situations, I remain committed to the view that they ought to be adopted by most of the human population at the present time. Provided that the ideas developed in the book gather significant political support, public health policy changes must be made that prohibit the consumption of animal products in many situations. I would like to thank my critics for engaging with my work and for providing me with the opportunity to clarify and expand on some ideas. It is my hope that this work will inspire others to read and engage with “Animal (De)liberation” and stimulate public health policy reform in accordance with this moral theory.
